# The Association Between Cognitive Deficits and Clinical Characteristic in First-Episode Drug Naïve Patients With Schizophrenia

**DOI:** 10.3389/fpsyt.2021.638773

**Published:** 2021-02-25

**Authors:** Xing-Jie Peng, Gang-Rui Hei, Ye Yang, Chen-Chen Liu, Jing-Mei Xiao, Yu-Jun Long, Jing Huang, Jing-Ping Zhao, Ren-Rong Wu

**Affiliations:** ^1^National Clinical Research Center for Mental Disorders, and Department of Psychiatry, The Second Xiangya Hospital of Central South University; National Technology Institute on Mental Disorders; Hunan Key Laboratory of Psychiatry and Mental Health, Changsha, China; ^2^Shanghai Institutes for Biological Sciences, Chinese Academy of Sciences, Shanghai, China

**Keywords:** cognitive impairment, first episode drug-naïve schizophrenia, MCCB, correlation analysis, positive and negative symptoms

## Abstract

**Background:** Schizophrenia is a severe mental disease which characterized by positive symptom, negative symptom, general pathology syndrome and cognitive deficits. In recent years, many studies have investigated the relationship between cognitive deficits and clinical characteristics in schizophrenia, but relatively few studies have been performed on first-episode drug-naïve patients.

**Methods:** Eighty seven first-episode drug-naïve schizophrenia patients were assessed for positive symptom, negative symptom, general pathology symptom and cognitive deficits from the Positive and Negative Symptom Scale and MATRICS Consensus Cognitive Battery. Psychotics depression were assessed using the Calgary depressing scale for schizophrenia. The relationship between clinical characteristics and cognitive deficits were assessed using correlation analysis and linear regression analysis.

**Results:** The prevalence of cognitive deficits among the patients in our study was 85.1% (74/87) which was much higher than that in the general population. According to correlation analysis, negative symptom was negatively correlated with speed of processing and social cognition, and general pathology showed a negative correlation with attention/vigilance. In addition, a positive correlation was found between age and speed of processing. No correlation was found between cognitive deficits and positive symptom.

**Conclusions:** This study confirmed that negative symptom is negatively related with some domains of cognitive function in first-episode drug naïve schizophrenia patients.

**Trail Registration:** NCT02880462. Registered August 26, 2016.

## Background

Schizophrenia is a severe mental disease with a poor prognosis, which is characterized by positive symptoms, negative symptoms, general pathology symptoms and multiple cognitive deficits (1, 2). These cognitive defects are found throughout the illness, both in the prodromal state before the onset of the full-blown disease and persisting even after symptomatic remission (3–5). Currently, first- and second-generation antipsychotic medications are the mainstay treatment for schizophrenia, which have great improvement of positive symptoms but have little effect on cognitive deficits (6–9). Moreover, cognitive deficits are associated with poor performance on social and personal functions, leading to a poor prognosis (10–12). Therefore, exploring the potential indicator of cognitive deficits is meaningful for timely intervention and reasonable assessment of prognosis in schizophrenia.

In recent years, some studies have reported that traditional clinical symptoms including positive symptoms, negative symptoms, general pathology symptoms are related to cognitive deficits in schizophrenia (13–16). Of the chronic schizophrenia, cross-section research has found that cognitive deficits are related to negative symptoms, especially deficits on executive function, verbal memory, and visual memory (17–19). While majority of such studies have found no relationship between positive symptoms and cognitive deficits. These reports suggest that clinical symptoms, especially negative symptoms, may be feasible indicators for predicting the cognitive deficits in schizophrenia. However, a few number of investigators have reached inconsistent results. They have reported a correlation between positive symptoms and cognitive deficits, and have displayed no correlation between negative symptoms and cognitive deficits (20). The different results can be explained due to different antipsychotics treatment, choice of cognitive measures, different clinical stages, modern electroconvulsive therapy, small sample and so on. Accordingly, in order to eliminate the potential factors, studies in first episode drug-naïve schizophrenia are required to discover the relationship between clinical symptoms and cognitive deficits. At present, there are only few studies on first episode drug-naïve patients.

Therefore, the aim of the present study is to identify the relationship between cognitive deficits and clinical symptoms. Measurement and Treatment Research Improve Cognition in Schizophrenia (MATRICS) is thought to be a representative test for schizophrenia cognitive assessment and has been widely used in China to assess cognitive function. Clinical symptoms are evaluated by Positive and Negative Symptoms Scale (PANSS). The hypothesis of the present study is that cognitive deficits are correlated to negative symptom but not correlated to positive symptoms in first episode drug-naïve schizophrenia patients.

## Method

### Participants

A total of 87 first-episode drug-naïve patients with schizophrenia admitted to four hospitals in different Chinese provinces from December 2016 to May 2019 were included in the study. Using procedures were approved by the ethical standards of the Committee on Human Experimentation of local institution, written informed consent was obtained from all participants or their substitute decision maker prior to be included in this study.

At the time of enrollment of the study, participants were between the age of 18 and 50 years with a first psychotic episode of schizophrenia, whose duration of illness is <36 months. Participants had never taken antipsychotics before. Patients who were diagnosed with schizophrenia in accordance with criteria set out in the Diagnostic and Statistical Manual of Mental Disorders Fourth Edition (DSM-IV). Patients who met any of the following criteria were excluded from our study: (1) included history of substance dependence defined by DSM-IV; (2) organic brain disorder; (3) high risk of suicide; (4) history of used Modified Electroconvulsive Therapy (MECT); (5) co-morbidity serious physical disorder; (6) pregnant or lactating women; and (7) history of treatment with antidepressant, mood stabilizers or other psychiatric drugs in last 3 months.

### Clinical Measures

The general clinical characteristics of the patients was assessed by two psychiatrists with rich experience by using the Positive and Negative Symptom Scale (PANSS) and the Calgary Depressing Scale for Schizophrenia (CDSS). The PANSS is composed of positive, negative and general psychopathology subscales. The PANSS subscales scores and CDSS were used for further correlation analysis.

### Cognitive Assessment

The MATRICS consensus cognitive battery (MCCB) is used to assess the cognitive function of patients, and is considered to have minimum practice effects (21). The MCCB consists of nine standardized cognitive tests which reflects seven domains of cognitive function, including the speed of processing [trail making test: part A (TMT), brief assessment of cognition in schizophrenia: symbol coding (BACS SC), and category fluency: animal (Animal fluency)], attention/vigilance [Continuous Performance Test (CPT)], working memory [digital sequence and Wechsler memory scale spatial span (WMS III)], verbal learning [Hopkins verbal learning test-revised (HVLT-R)], visual learning [brief visuospatial memory test-revised (BVMT-R)], reasoning/problem solving [neuropsychological assessment battery (NAB)], and social cognition [Mayer–Salovey–Caruso emotional intelligence test (MSCEIT)]. In the last few years, the MCCB has been translated into Chinese and was widely used in both healthy individuals and schizophrenia patients (22, 23). All cognitive assessments were performed in strict accordance with the instruction manual of the tools by trained personnel. All test scores were adjusted according to Chinese standards for age, gender and education level, and converted into T-scores. The Global Deficit Score (GDS) was used to identify the severity degree of cognitive dysfunction of patients with schizophrenia. All the MCCB raw subtest scores were translated into T-scores and converted to deficit scores (DS) according to the following criteria: *DS* = 0 when T-score > 39 (normal cognitive function), *DS* = 1 as 35 ≤ T-score ≤ 39 (mild impairment), *DS* = 2 as 30 ≤ T-score ≤ 34 (mild to moderate impairment), *DS* = 3 as 25 ≤ T-score ≤ 29 (moderate impairment), *DS* = 4 as 20 ≤ T-score ≤ 24 (moderate to severe impairment), and *DS* = 5 as T-score <20 (severe impairment). The final GDS was defined as the average of the DS obtained from each MCCB raw subtest scores. Individuals with a final GDS of ≥0.5 were defined as having cognitive impairment (24, 25).

### Statistical Analysis

All analyses were conducted in SPSS, version 26.0. Beyond descriptive analysis, data were tested for normality of distribution by using the Shapiro-Wilk test. The relationship between clinical characteristics and cognitive function were analyzed in two steps. In step 1, two-tailed Pearson's correlation analysis and Spearman correlation analysis were used to determine the preliminary association between cognitive measures with symptom dimension scores. In step 2, linear regressions were used to determine the relationships which showed statistical significance in step 1, after control for the potentially confounding variables, i.e., age, BMI, duration of illness, and education years and smoking. The statistical significance was set as *p* < 0.05.

## Results

### Demographic and Clinical Characteristic

A total of 87 patients were included in our study. Table 1 summarizes the demographics and clinical characteristics of the patients. The average age of patients included in our study was 24.22 years [standard deviation (SD), 6.84; range. 18–47 years]. Thirty six patients (41.38%) subjects were male, and 7 patients had smoking history. The average of illness duration was 10.17 months (SD, 10.33; range 1–36 months). The average year of education was 11.08 (SD, 2.57) and BMI (body mass index) was 21.10 (SD, 3.58) kg/m^2^.

**Table 1:** Demographic and clinical characteristics of full sample (*N* = 87).

	***N*** **=** **87**	
	***N***	**%**
Male	36	41.38
Smoking	7	8.05
	**Mean**	**SD**
Age	24.22	6.84
BMI	21.10	3.58
Education (years)	11.08	2.57
Illness duration (months)	10.17	10.33
Total PANSS	98.36	13.70
PANSS P	23.54	6.39
PANSS N	24.94	6.91
PANSS G	49.87	7.35
CDSS	3.53	3.41

*BMI, body mass index; PANSS P, positive and negative symptom scale positive score; PANSS N, positive and negative symptom scale negative score; PANSS G, positive and negative symptom scale general pathology score; Total PANSS, positive and negative symptom scale total score; CDSS, Calgary depressing scale for schizophrenia*.

The average PANSS total scores for study patients on admission were 98.36 (SD, 13.70) among which PANSS positive scores were 23.54 (SD, 6.39), PANSS negative scores were 24.94 (SD, 6.91) and PANSS general pathology scores were 49.87 (SD, 7.35). The average scores of CDSS were 3.53 (SD, 3.53) with a range from 0 to 15.

### Cognitive Function

Table 2 shows the MCCB standardized domain scores and raw subtest scores of the patients. Compared to the T-scores of standard cognitive performances (*M*, 50; SD, 10) of 656 healthy adults in China, patients in our study showed significant cognitive deficits (all *p* < 0.001, see Supplementary Table 1). According to the GDS criteria on cognitive impairment, 74/87 (85.1%) of patients had cognitive impairment on clinical neuropsychological tests, and it was much higher than that in general population in China (16.5%).

**Table 2:** MCCB standardized domain scores and raw subtest scores of full samples (*N* = 87).

	***N*** **=** **87**	
	**Mean**	**SD**
**MCCB domain (** ***T*** **-scores)**		
Speed of processing	35.11	9.12
Working memory	38.00	12.36
Reasoning/problem solving	39.41	12.96
Verbal learning	34.80	10.66
Visual learning	39.97	13.57
Attention/vigilance	38.97	11.93
Social cognition	38.13	12.00
**MCCB subtest (raw scores)**		
Trails part A	36.91	12.19
BASC symbol coding	31.25	11.43
HVLT-R total score	34.80	10.66
BVMT-R total score	39.97	13.57
Category fluency (animals)	37.16	12.14
CPT	38.97	11.93
MSCEIT managing emotions	38.13	12.00
WMS spatial span	38.00	12.36
NAB mazes	39.41	12.96

*Domain scores are presented as T-scores (M, 50; SD, 10) and domain subtest scores are presented as raw data scores (e.g., number of seconds to complete Trails part A). Trails part A, trail making test: part A; BASC symbol coding, brief assessment of cognition in schizophrenia: symbol coding; HVLT-R total score, Hopkins verbal learning test-revised; WMS spatial span, digital sequence and Wechsler memory scale spatial span; NAB mazes, neuropsychological assessment battery; BVMT-R total score, brief visuospatial memory test-revised; CPT, continuous performance test; Category fluency (animals), category fluency: animal; MSCEIT managing emotions, Mayer–Salovey–Caruso emotional intelligence test*.

### Correlation Between PANSS and Cognitive Test Scores

**Figure 1:**
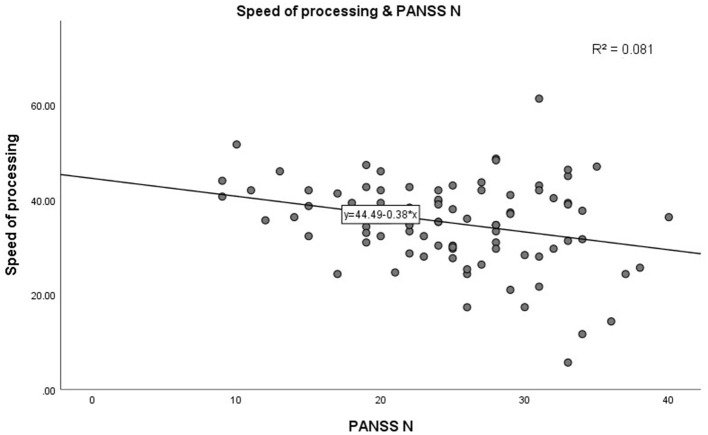
Correlation between PANSS N and speed of processing.

**Figure 2:**
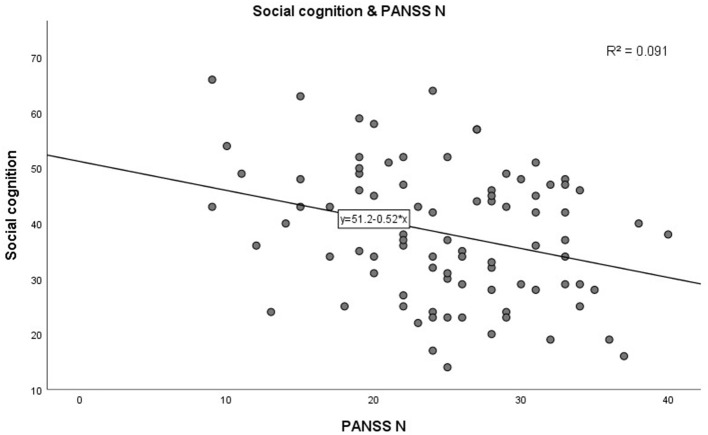
Correlation between PANSS N and social cognition.

**Figure 3:**
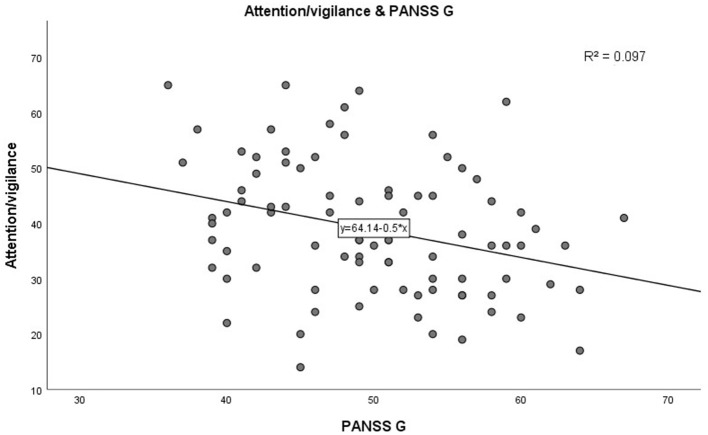
Corrleation between PANSS G and Attention/vigilance.

In step 1, as shown in Table 3. PANSS negative scores were significantly negatively correlated with the MCCB speed of processing domain (*r* = −0.285, *p* = 0.007, see Figure 1). A similar trend was noted toward a negative association between PANSS negative scores and the MCCB social cognition domain (*r* = −0.302, *p* = 0.004, see Figure 2). In addition, PANSS general pathology scores were significant negatively correlated with the MCCB attention/vigilance domain (*r* = −0.311, *p* = 0.003, see Figure 3). Lastly, PANSS total scores were negative associated with the MCCB attention/vigilance domain scores (*r* = −0.332, *p* = 0.002). No significant association was found between other MCCB domain scores and PANSS raw subtest scores, and no association was found between CDSS scores and MCCB domain scores.

**Table 3:** Correlation between clinical characteristics and cognitive dysfunction.

	**PANSS total scores**		**PANSS P**		**PANSS N**		**PANSS G**		**CDSS**	
	***r***	***p***	***r***	***P***	***r***	***P***	***r***	***p***	***r***	***p***
Speed of processing	−0.108	0.321	0.183	0.089	−0.285	0.007**	−0.096	0.376	0.029	0.788
Working memory	−0.175	0.106	−0.033	0.759	−0.109	0.317	−0.192	0.074	−0.141	0.192
Reasoning/problem solving	−0.041	0.709	−0.076	0.483	0.015	0.890	−0.070	0.519	0.064	0.558
Verbal learning	−0.062	0.570	0.083	0.447	−0.134	0.217	−0.028	0.794	0.058	0.595
Visual learning	0.186	0.085	0.079	0.468	0.074	0.496	0.140	0.197	0.039	0.717
Attention/vigilance	−0.332	0.002**	−0.103	0.341	−0.206	0.055	−0.311	0.003**	−0.046	0.670
Social cognition	−0.184	0.087	0.160	0.138	−0.302	0.004**	−0.211	0.050	−0.014	0.898

*The correlation between CDSS and cognitive measures is used Spearman analysis because the distribution of CDSS is non-normal. Others are used Pearson analysis*.

Note: *p < 0.05, **p < 0.01.

### Linear Regression Analysis

Further linear regression analysis is shown in Table 4. In step 2, linear regression models were constructed to assess the relationship between clinical characteristics and cognitive test scores. Demographic characteristics (age, gender, BMI, duration of illness, education years, and smoking) factors were included as covariates. Increase in PANSS negative scores (β = −0.347, *p* = 0.001) significantly predicted high speed of processing performance, adjusting for age, gender, BMI, smoking, duration of illness, and education years. Interestingly, there was a significant increase in speed of processing with age (β = 0.428, *p* < 0.001). In addition, increase in PANSS negative scores predicted worse social cognition (β = −0.327, *p* = 0.005) after adjusting for covariates. Moreover, PANSS general pathology scores had a significant negative correlation with attention/vigilance (β = −0.259, *p* = 0.021) after adjusting for potential confounding factors. No significant correlation between the remaining cognitive test scores and the PANSS score and demographic characteristics, was found.

**Table 4:** Results of the regression analysis.

	**Regression**															
	**PANSS N**		**PANSS G**		**Age (years)**		**Gender**		**BMI**		**Duration of illness**		**Education years**		**smoking**	
	**β**	***P***	**β**	***P***	**β**	***P***	**β**	***P***	**β**	***P***	**β**	***P***	**β**	***P***	**β**	***P***
Speed of processing	−0.347	0.001**	–	–	0.428	<0.001***	0.140	0.173	−0.084	0.447	−0.094	0.347	−0.049	0.664	0.117	0.263
Attention/vigilance	–	–	−0.259	0.021*	−0.150	0.227	−0.007	0.951	0.014	0.911	−0.024	0.824	−0.028	0.811	0.111	0.339
Social cognition	−0.327	0.005**	–	–	0.148	0.225	0.099	0.374	−0.118	0.330	0.110	0.313	−0.016	0.896	−0.049	0.669

*p < 0.05, **p < 0.01, ***p < 0.001.

## Discussion

This study tried to determine the association between cognitive impairment and clinical characteristics in first-episode drug-naïve patients in schizophrenia, and yielded 3 main findings. First, first-episode drug-naïve schizophrenia patients have severe cognitive impairment. Second, the negative symptom and the general symptomatology are negatively correlated with some domains of cognitive impairment in first-episode drug-naïve schizophrenia patients, but there is no association between positive symptom and cognitive impairment. Third, the age is positively correlated with the speed of processing, it suggests that older patients may have better speed of processing than younger in first-episode drug naïve schizophrenia.

Results from this study confirmed our hypothesis that the first-episode drug-naïve schizophrenia patients have severe cognitive impairment. This finding is consistent with some previously studies published before, which reported that patients had cognitive impairment at the time of first diagnosis (26–28). Interestingly, in recent years, some studies have reported that in the prodromal period of schizophrenia, some patients have cognitive impairment, and this group of patients have a higher proportion to develop schizophrenia compared with patients without cognitive impairment in the prodromal stage (29). And cognitive defects are progressively worse with the disease progresses from prodromal stage to schizophrenia (30). This finding suggests that cognitive deficits in schizophrenia appear very early in the onset of schizophrenia, and cognitive deficits may be the outcome of abnormal development of the brain, leading to problems in acquiring cognitive abilities.

The results of this study support that cognitive function is not related to positive symptom. Some studies have reached similar conclusions, though a few studies have drawn contrary conclusions. Currently, antipsychotics including the first-generation antipsychotics and the second-generation antipsychotics are the main therapies for schizophrenia. Though antipsychotics have a promising therapeutic effect for positive symptoms, effects of antipsychotics on cognitive function remains dispute. Results of our study suggest that cognitive function is an independent syndrome of positive symptom in schizophrenia, indicating different potential pathogenesis. And this may be the root cause for limited effect of antipsychotics on cognitive function. Moreover, results of our study suggest that speed of processing and social cognition are negative correlated with negative symptom, and attention/vigilance is negative correlated with general symptomatology in first-episode drug naïve schizophrenia patients. Speed of processing seems to be impaired already in the early stages of emerging psychosis and has previously been suggested as a possible predictor for transition to psychosis (31). One possible reason for deficits in speed of processing is that the prefrontal cortex GABA hypofunction in schizophrenia patients (32). Furthermore, the associations between negative symptoms and social cognition are reported in previous studies (33, 34). Interestingly, some studies have also reported the predicative effect of social cognition. Social cognitive deficits can act as an important endophenotype for estimating the risk of schizophrenia in at risk siblings (35). In addition, similar results in association between negative symptoms and cognitive function have been found in other studies. Some previous studies have shown that negative symptoms are negatively associated with both immediate memory and language indexes in first-episode schizophrenia (36). However, distinct results in the relationship between negative symptom and different domains of cognitive function were found. This inconsistency may be affected by duration of illness, antipsychotics, cognitive measurement, samples in different studies. In recent years, some reports have also suggested that psychotic depression can also affect cognitive function (37). However, no correlation was found between psychotic depression and cognitive deficits in our study. Overall, we confirmed that no correlation between positive symptom and cognitive deficits and a negative correlation between negative symptom and cognitive function in first-episode drug-naïve schizophrenia patients, and negative symptom and cognitive deficits may share a common mechanism. Further studies should be implemented to explore the potential mechanism.

Moreover, speed of processing was inversely associated with age in our study. There were two possible explanations for this result. First, the age of patients in our study were ranged from 18 to 47 years, and this development of speed of processing might owing to richer social experience in older patients. Second, patients included in this study are first-episode drug-naïve patients whose duration are <36 months. So, this result may owe to the late onset of illness. In the previous studies have shown that the earlier onset was, the more serious the cognitive function would be (38, 39). And this would also provide the theory basis for the second explanation. Plus, recent years, cognitive function was considered to be a stable marker in duration of schizophrenia (40). So, finding of our study suggested a correlation between cognitive function and negative symptoms in first-episode drug naïve schizophrenia patients. However, since our research is a cross-sectional study, the further follow-up studies should be down to examine the changes in cognitive function over time.

There are some limitations to be noted in our study. First, the cross-sectional design of this study failed to examine the temporal relationship between cognitive function and clinical characteristics. Second, we did not collect healthy controls for the comparison of the cognitive function of patients in our study. Third, our study was performed in a Chinese Han population, and the data should be extrapolated to other regions and ethnic groups cautiously.

## Conclusions

In summary, this study confirmed that severe cognitive deficits appear early in the disease of schizophrenia. Moreover, cognitive deficits in first-episode drug-naïve schizophrenia patients are not correlated to positive symptom, but correlated to negative symptom and general pathology syndrome. In addition, speed of processing is inversely associated with age in this study. It suggests that older patients may have better speed of processing than younger in first-episode drug naïve schizophrenia. No correlation between psychotic depression and cognitive deficits are found. Considering the limitations of our study, well-designed longitudinal studies are needed in the future to verify our conclusions.

## Data Availability Statement

The raw data supporting the conclusions of this article will be made available by the authors, without undue reservation.

## Ethics Statement

The studies involving human participants were reviewed and approved by the Second Xiangya Hospital Ethics Committee. The patients/participants provided their written informed consent to participate in this study.

## Author Contributions

X-JP analyzed and interpreted the patient data and was a major contributor in writing the manuscript. G-RH mainly designed and performed the study. YY, C-CL, J-MX, Y-JL, and JH helped in patient recruitment, monitor of the data quality, and document treatment emergent adverse events. J-PZ guided the study design. R-RW was responsible for the overall content. All authors read and approved the final manuscript.

## Conflict of Interest

The authors declare that the research was conducted in the absence of any commercial or financial relationships that could be construed as a potential conflict of interest.

## Abbreviations

PANSS: positive and negative syndrome scale
DSM-IV: diagnostic and statistical manual of mental disorders fourth edition
MECT: modified electroconvulsive therapy
CDSS: Calgary depressing scale for schizophrenia
TMT: trail making test
part A, BACS SC: brief assessment of cognition in schizophrenia symbol coding
CPT: continuous performance test
WMS III: digital sequence and Wechsler memory scale spatial span
HVLT-R: Hopkins verbal learning test-revised
BVMT-R: brief visuospatial memory test-revised
NAB: neuropsychological assessment battery
MSECIT: Mayer–Salovey–Caruso emotional intelligence test
GDS: global deficit score
DS: deficit score.
