# Corrigendum: The Association Between Cognitive Deficits and Clinical Characteristic in First-Episode Drug Naïve Patients With Schizophrenia

**DOI:** 10.3389/fpsyt.2021.678836

**Published:** 2021-04-23

**Authors:** Xing-Jie Peng, Gang-Rui Hei, Ye Yang, Chen-Chen Liu, Jing-Mei Xiao, Yu-Jun Long, Jing Huang, Jing-Ping Zhao, Ren-Rong Wu

**Affiliations:** ^1^National Clinical Research Center for Mental Disorders, and Department of Psychiatry, The Second Xiangya Hospital of Central South University; National Technology Institute on Mental Disorders; Hunan Key Laboratory of Psychiatry and Mental Health, Changsha, China; ^2^Shanghai Institutes for Biological Sciences, Chinese Academy of Sciences, Shanghai, China

**Keywords:** cognitive impairment, first episode drug-naïve schizophrenia, MCCB, correlation analysis, positive and negative symptoms

In the published article, there was an error in affiliations 1,2,3,4, and 5. Instead of “1 Mental Health Institute of the Second Xiangya Hospital, Central South University, Changsha, China, 2 China National Clinical Research Center on Mental Disorders, Changsha, China, 3 China National Technology Institute on Mental Disorders, Central South University, Changsha, China, 4 Hunan Key Laboratory of Psychiatry and Mental Health, Chinese Academy of Sciences, Changsha, China, 5 Shanghai Institutes for Biological Sciences, Chinese Academy of Sciences, Shanghai, China,” it should be “1 National Clinical Research Center for Mental Disorders, and Department of Psychiatry, The Second Xiangya Hospital of Central South University; National Technology Institute on Mental Disorders; Hunan Key Laboratory of Psychiatry and Mental Health, Changsha, China, 2 Shanghai Institutes for Biological Sciences, Chinese Academy of Sciences, Shanghai, China.”

The authors apologize for this error and state that this does not change the scientific conclusions of the article in any way. The original article has been updated.

