# Supplementary material for: The Association Between Cognitive Deficits and Clinical Characteristic in First-Episode Drug Naïve Patients With Schizophrenia
Source: Front Psychiatry. 2021 Feb 25;12:638773. doi: 10.3389/fpsyt.2021.638773 (PMC7950319; doi:10.3389/fpsyt.2021.638773)
Supplement: Supplementary file 1 [file Data_Sheet_1.docx]

**Supplementary**

Table S1. Cognitive function in first-episode drug-naive schizophrenia and normal

Table S2. Comparing the general clinical characteristics and cognitive scores in female and male patients.

Table S1. Cognitive function in first-episode drug-naive schizophrenia and normal

|  | Patients | | Controls ^s^ | | P value |
| --- | --- | --- | --- | --- | --- |
|  | *N* = 87 | | *N* = 656 | |  |
|  | Mean | SD | Mean | SD |  |
| MCCB domain (*T* scores) |  | |  | |  |
| Speed of processing | 35.11 | 9.12 | 50.00 | 10.00 | <0.001*** |
| Working memory | 38.00 | 12.36 | 50.00 | 10.00 | <0.001*** |
| Reasoning/problem solving | 39.41 | 12.96 | 50.00 | 10.00 | <0.001*** |
| Verbal learning | 34.80 | 10.66 | 50.00 | 10.00 | <0.001*** |
| Visual learning | 39.97 | 13.57 | 50.00 | 10.00 | <0.001*** |
| Attention/vigilance | 38.97 | 11.93 | 50.00 | 10.00 | <0.001*** |
| Social cognition | 38.13 | 12.00 | 50.00 | 10.00 | <0.001*** |
| MCCB subtest (raw scores) |  | |  | |  |
| Trails part A | 36.91 | 12.19 | 50.00 | 10.00 | <0.001*** |
| BASC symbol coding | 31.25 | 11.43 | 50.00 | 10.00 | <0.001*** |
| HVLT-R total score | 34.80 | 10.66 | 50.00 | 10.00 | <0.001*** |
| BVMT-R total score | 39.97 | 13.57 | 50.00 | 10.00 | <0.001*** |
| Category fluency (animals) | 37.16 | 12.14 | 50.00 | 10.00 | <0.001*** |
| CPT | 38.97 | 11.93 | 50.00 | 10.00 | <0.001*** |
| MSCEIT managing emotions | 38.13 | 12.00 | 50.00 | 10.00 | <0.001*** |
| WMS spatial span | 38.00 | 12.36 | 50.00 | 10.00 | <0.001*** |
| NAB mazes | 39.41 | 12.96 | 50.00 | 10.00 | <0.001*** |
| GDS |  | |  | |  |
| GDS < 0.50  0.5 < GDS ≤ 1  1 < GDS ≤ 2  2 < GDS ≤ 3  3 < GDS ≤ 4  4 < GDS ≤ 5  GDS ≥ 5 | 16.1%  23.0%  39.1%  13.8%  6.9%  1.1%  0% | | 83.5%  13.6%  2.6%  0.3%  0%  0%  0% | |  |
| Note: ^S^ Normative standards of China were based on Shi et al., 2015 published before which included 656 healthy controls.  *p < 0.05 **p < 0.01 ***p < 0.001 | | | | | |

**Table S2.** **Comparing the general clinical characteristics and cognitive scores in female and male patients.**

|  | Male  *N* = 36 | | Female  *N* = 51 | | t/ X^2^ | P |
| --- | --- | --- | --- | --- | --- | --- |
|  | Mean | SD | Mean | SD |  | |
| Age | 24.06 | 7.06 | 24.33 | 6.75 | -0.185 | 0.853 |
| BMI | 21.71 | 3.43 | 20.68 | 3.66 | 1.330 | 0.187 |
| Education (years) | 10.36 | 2.27 | 11.59 | 2.66 | -2.248 | 0.027* |
| Illness duration (months) | 11.63 | 11.30 | 9.15 | 9.57 | 1.107 | 0.272 |
| Total PANSS | 97.61 | 12.51 | 98.88 | 14.58 | -0.424 | 0.672 |
| PANSS P | 22.03 | 5.74 | 24.61 | 6.66 | -1.882 | 0.063 |
| PANSS N | 26.11 | 6.11 | 24.12 | 7.38 | 1.330 | 0.187 |
| PANSS G | 49.47 | 7.66 | 50.16 | 7.19 | -0.426 | 0.671 |
| TMT | 36.25 | 12.72 | 37.37 | 11.90 | -0.421 | 0.675 |
| BACS SC | 27.33 | 11.42 | 34.02 | 10.70 | -2.792 | 0.006** |
| HVLT-R | 33.00 | 11.17 | 36.08 | 10.21 | -1.333 | 0.186 |
| WMS III | 41.00 | 12.51 | 35.88 | 11.93 | 1.932 | 0.057 |
| NAB | 44.89 | 13.58 | 35.55 | 11.08 | 3.525 | 0.001*** |
| BVMT-R | 38.50 | 14.70 | 41.00 | 12.76 | -0.845 | 0.401 |
| CPT | 39.64 | 12.05 | 38.49 | 11.95 | 0.440 | 0.661 |
| Animal fluency | 35.64 | 12.03 | 38.24 | 12.22 | -0.983 | 0.329 |
| MSCEIT | 35.89 | 10.85 | 39.71 | 12.62 | -1.471 | 0.145 |
| Speed of processing | 33.07 | 8.49 | 36.54 | 9.35 | -1.769 | 0.080 |
| Attention/Vigilance | 39.64 | 12.05 | 38.49 | 11.95 | 0.440 | 0.661 |
| Working and memory | 41.00 | 12.51 | 35.88 | 11.93 | 1.932 | 0.057 |
| Verbal learning/Memory | 33.00 | 11.17 | 36.08 | 10.21 | -1.333 | 0.186 |
| Visual learning/Memory | 38.50 | 14.70 | 41.00 | 12.76 | -0.845 | 0.401 |
| Reasoning/Problem solving | 44.89 | 13.58 | 35.55 | 11.08 | 3.525 | 0.001*** |
| Social cognition | 35.89 | 10.85 | 39.71 | 12.62 | -1.471 | 0.145 |
| *Note:*  TMT= trail making test: part A; BASC SC= brief assessment of cognition in schizophrenia: symbol coding; HVLT-R= Hopkins verbal learning test-revised; WMS III= digital sequence and Wechsler memory scale spatial span; NAB= neuropsychological assessment battery; BVMT-R= brief visuospatial memory test-revised; CPT= continuous performance test; animal fluency= category fluency: animal; MSCEIT= Mayer–Salovey–Caruso emotional intelligence test.  PANSS P: Positive and negative symptom scale positive score;  PANSS N: Positive and negative symptom scale negative score;  PANSS G: Positive and negative symptom scale General Pathology score;  Total PANSS: Positive and negative symptom scale total score.  *p < 0.05 **p < 0.01 ***p < 0.001 | | | | | | |
